# Improving Pathological Assessment of Breast Cancer by Employing Array-Based Transcriptome Analysis

**DOI:** 10.3390/microarrays2030228

**Published:** 2013-08-29

**Authors:** Zsuzsanna Mihály, Balázs Győrffy

**Affiliations:** 1 1st Deptartment of Pediatrics, Semmelweis University, Budapest H-1083, Hungary; E-Mail: zsuzsannamihaly@gmail.com; 2 Research Laboratory of Pediatrics and Nephrology, Hungarian Academy of Sciences, Budapest H-1083, Hungary

**Keywords:** breast cancer, microarray, molecular subtype, prognosis, prediction, biomarker

## Abstract

Breast cancer research has paved the way of personalized oncology with the introduction of hormonal therapy and the measurement of estrogen receptor as the first widely accepted clinical biomarker. The expression of another receptor—HER2/ERBB2/neu—was initially a sign of worse prognosis, but targeted therapy has granted improved outcome for these patients so that today HER2 positive patients have better prognosis than HER2 negative patients. Later, the introduction of multigene assays provided the pathologists with an unbiased assessment of the tumors’ molecular fingerprint. The recent FDA approval of complete microarray pipelines has opened new possibilities for the objective classification of breast cancer samples. Here we review the applications of microarrays for determining ER and HER2 status, molecular subtypes as well as predicting prognosis and grade for breast cancer patients. An open question remains the role of single genes within such signatures. Openly available microarray datasets enable the execution of an independent cross-validation of new marker and signature candidates. In summary, we review the current state regarding clinical applications of microarrays in breast cancer molecular pathology.

## 1. Introduction

Today, pathological evaluation is still a gold standard for breast cancer diagnosis. Besides the routine assessment it includes the investigation of three molecular markers: estrogen receptor (ESR1), progesterone receptor (PGR) and HER2 receptor. The expression of ESR1 is used to predict response to endocrine therapies, which deliver improved outcome for ESR1 positive patients while no response can be expected in ESR1 negative patients [1]. We must note that only half of ESR1 positive patients will actually benefit from endocrine therapy [2]. PGR, a gene regulated by ESR1, can be used as a surrogate marker for ESR1 status determination. HER2 is used to predict response to trastuzumab, and—similarly to ESR1—about half of HER2 positive patients will respond to anti-HER2 therapy [3]. HER2 positivity was once a sign of worse prognosis, but since the introduction of targeted therapy the expectations have changed: today patients with HER2 positive disease can actually expect a better survival than those with a negative cancer [4]. These markers are also termed negative biomarkers, because they can only be employed to predict the lack of response.

At the same time, there are numerous studies questioning the reliability of pathological evaluation. The assessment of ESR1 expression is significantly different among various laboratories [5]. When comparing laboratories from 26 countries, the results for ESR1 IHC were erroneous in up to 30–60% of the analyses [6]. In another comparison of 105 laboratories by an external quality assessment, only 36% delivered reliable results [7]. Although the reproducibility was good for all antibodies used for ESR1 status determination, the different ESR1 antibodies and heat-induced epitope retrieval could influence the final determination of ESR1-positivity [8]. The interobserver agreement in HER2 status determination was questioned by comparing the diagnosis of nine pathologists for the same sample [9]. Similar discrepancies were observed for tumor grade: concordant vote by three pathologists was only 43% [10]. When comparing the diagnoses of two independent pathologists in 405 node negative patients, a discrepancy of 20% was observed [11].

These results were the main driving force behind the development of the first multigene assays enabling an unbiased determination of pathological parameters. The high concordance between microarrays and RT-PCR [12] and the correlation between different microarray platforms [13] has been documented previously. Moreover, the application of unambiguous mapping between genes and probes within one array platform enables the consistent and reliable measurement of gene expression [14]. Based on these observations, microarrays promised an optimal alternative strategy to acquire the diagnoses needed in the pathological determination of breast cancer biomarkers.

## 2. The Discovery of Molecular Subtypes

It has been established that the IHC-based analysis of classical markers can define new subcategories [15] which can differ not only in the proper diagnosis but also in prognosis. In 2001, the intrinsic molecular subtypes including luminal A, luminal B, luminal C, basal-like and HER2 positive cohorts of breast cancer were proposed by Sorlie *et al.* [16]. After validation of the subtypes in three independent gene-sets, the molecular subtypes were settled to luminal A, luminal B, basal-like, HER2 positive and normal-breast-like [17]. Since response to various systemic therapies differs tremendously among these molecular subgroups, the clinical decision making for the appropriate therapy is also affected.

The molecular classification comprises two subtypes (luminal A and B) for ESR1 positive tumors. These luminal subtypes express cytokeratins 8/18, ESR1 and genes associated with an active ESR1 pathway. The luminal A subtype expresses low proliferation rates and it is associated with good prognosis contrary to luminal B, which has high proliferation rates and higher histological grade with worse prognosis. When comparing all four subtypes in over 2,000 patients, luminal A tumors had the lowest rate of relapse while luminal B, HER2 positive, and basal-like subtypes were associated with an increased risk of relapse [18]. In an independent analysis of the patient samples originally used by Sorlie *et al.* higher drug sensitivity among luminal A patients was suggested to provide the basis for better patients survival as compared to luminal B patients [19].

Tumors of the HER2 positive subtype overexpress HER2 and genes associated with the HER2 amplicon and the HER2 pathways but they are ESR1 negative. Basal-like tumors can be characterized by cytokeratin 5, 17, caveolin 1 and 2, nestin, CD44 and EGFR expression and have the worst prognosis among all subtypes [20]. Furthermore, there is an overlap in definition between triple-negative breast cancer and the basal subtype due to the triple-negative profile of all basal samples [21].

Meanwhile a study warned that identification of luminal cancers and normal breast-like cancers by visual inspection of dendrograms obtained from hierarchical cluster analysis shows suboptimal levels of interobserver agreement while the identification of basal-like and HER2 cancers showed almost perfect interobserver agreement rates [22]. In contrast, a study validated the molecular subtypes in various microarray platforms and confirmed high reproducibility of the classification [23].

Although most of the classical histological types of breast cancer can be correlated to the molecular subtypes, the adenoid cystic and medullar carcinomas display basal-like signature despite the contradictory prognosis of the molecular and histological subtype [24]. Consequently, it seems that molecular subtypes might be divided into additional subgroups with further data from transcriptomic analyses. For example, the lack of ESR1 or PGR receptors in luminal A subtype can define new subgroups with unique clinicopathologic characteristics [25]. Further molecular markers are capable to estimate prognosis in a subtype-independent manner using claudin expression [26]. The subtypes have been extended by a “claudin low” group in which all five claudins display low expression [27]. The claudin-low subtype was a frequent phenomenon in metaplastic and basal-like breast cancer and was a strong predictor of disease recurrence [27]. Other data suggest subtype-specific differences in the relevance of proliferation-associated genes in addition to MKI67 [28].

In summary, the microarray-based characterization of the molecular subtypes can provide more detailed and individualized classification of the patients, making individual patient-tailored therapy possible. However, many of the additional biomarkers identified within the established subtypes have still to be converted from the transcriptomic data into guidelines. 

## 3. Determination of Receptor Status

Although IHC remains the gold standard for receptor status determination, a more reliable assessment using Affymetrix microarrays was established by Gong *et al.* [29]. In this, the receptor status determination is performed using Affymetrix HGU133x platforms by using a cutoff of 1,150 (MAS5 normalized value) for HER2 and 500 for ESR1. The ESR1 and HER2 mRNA expression quantification by microarrays correlate significantly with the corresponding clinical receptor status. Moreover, the array-based results are highly reproducible and are less influenced by the tissue acquisition method. In an analysis comparing microarrays and IHC, the arrays delivered a highly concordant, objective and quantitative assessment of tumor receptor status [30].

Although there is a high correlation between biomarker scoring by IHC and by gene expression, the gene expression determinations for ESR1 and ERBB2 status had higher prognostic power [31]. While the price of a microarrays has fallen in the past years, IHC or FISH still remain cheaper in the case of only four (HER2, ESR1, PGR, MKI67) examined markers.

However, the cost-effectiveness depends on the eligible number of biomarkers used to classify the patients regarding prognosis and therapy selection and the relative price of an array-based diagnostic test as such is lower when additional markers are included. By employing microarrays and/or IHC, a number of additional markers have been proposed for predicting response to hormonal therapy in breast cancer including MAPT [32], SLC7A5 [33], TP53 [33], DRG1 [33], CXCL10 [34], MT1 [35], and many more. These markers have been recently evaluated in two large pooled transcriptomic databases where PGR, MAPT and SLC7A5 have been identified as the most promising biomarkers for predicting survival after hormonal therapy [36].

In addition to single genes, epigenetic changes are also increasingly linked to cancer pathogenesis [37]. Among them is the reprogramming of the chromatin landscape which was also linked to endocrine resistance in breast cancer [38]. The development of resistance to endocrine therapy is a slow process involving extensive transcriptional reprogramming reminiscent of cell fate commitment [39]. However, in such scenarios the measurement of the entire transcriptome is not necessary as gene expression signatures of selected pathways can be representative for chromatin reprogramming as it was demonstrated for NOTCH-PBX1 activity [38].

In the near future simultaneous determination of ESR1 and HER2 status with additional marker genes will most probably be part of multigenic breast cancer classification. Even today there are multigene assays employing HER2 and ESR1 with a high significance in the applied scoring for endocrine-treated node-negative breast cancer patients [10]. In addition, an internet-based classification software is already available which is capable of deriving a hormone receptor status by an automated processing of commonly used commercially available genome-wide microarrays [40].

## 4. Prognostic Signatures

For specific clinico-pathological cohorts of breast cancer patients, new multigenic signatures developed using microarrays (MapQuant DX [41], Mammaprint [42]) and qPCR (Oncotype DX [10], Theros Breast Cancer Gene Expression Ratio Assay [43], PAM50 [44]) are already available as support tools for clinical decision-making.

Of these, the first one approved by the FDA as an *in vitro* diagnostic multivariate index assay (IVDMIA), was the 70-gene signature (Mammaprint) [42]. The 70-gene signature provides a risk prediction (low or high group) of distant recurrence after surgery in lymph node negative patients regardless of their ESR1 status or prior treatment [18]. In the case of node-negative patients, a prediction of low risk equals to a 13% risk of distant metastasis within 10 years while high risk equals to a 56% risk. Consequently, low risk patients may avoid chemotherapy. The scoring of the 70-gene signature was validated in the TRANSBIG trial [42] and in an independent study of the Massachusetts General Hospital [45].

Oncotype DX is a qPCR based test measuring the expression of 21 genes (including 5 housekeeping genes), which are used to calculate a recurrence score ranging between 1 and 100 [10]. The recurrence score provides a risk prediction of recurrence in 10 years for node negative, ESR1 positive patients [10]. A high recurrence score (over 30) is predictive for worse prognosis but it indicates a better response to chemotherapy. The test was also validated in independent clinical studies [46,47,48]. The reproducibility of qPCR-based results was also confirmed by employing Affymetrix microarrays [40]. However, genes have various weights in the analysis and known clinical parameters (ESR1, HER2) have the highest contribution to the final score. Without the application of these weights, the expression signature of the 21 genes is not capable of predicting survival as it was demonstrated in 1,079 breast cancer patients [49]. In addition, when assessing concordance between Oncotype DX results and IHC/FISH, an unacceptably high false negative rate (58%) of Oncotpye DX was observed while all patients designated by Oncotype DX as HER2 positive were also positive by IHC/FISH [50].

To date, the remaining tests have gained limited clinical use. MapQuant DX signature evaluates 98 genes in a molecular diagnostic test for estrogen receptor positive, grade II breast cancer patients to measure tumor proliferation, the risk of metastasis and response to chemotherapy [41]. The H:I ratio, also known as Theros Breast Cancer index is a molecular grade index for ESR1-positive breast cancer patients treated with tamoxifen [43]. It can stratify tamoxifen-treated and untreated breast cancer patients into high and low risk of recurrence which cohorts differ in outcome within 5 years. The H:I ratio was also evaluated in additional cohorts of patients [51,52], however discrepant results were documented in a different study [53]. The Invasive Gene Signature (IGS) [54] and the HER2-Drived Prognostic Predictor (HDP) [55] are based on microarray assays, while the Celera Metastasis Score [56] and the BreastOnc DX [57] are PCR based.

We must note here the requirement to adequately identify molecular subtypes of breast cancer which is not achieved by routine IHC panel alone. The qPCR based PAM50—Breast Bioclassifier—test can define these subtypes from FFPE [44] and can therefore provide estimation of prognosis as well [58]. In addition, it is capable of predicting a response to neoadjuvant endocrine therapy of ER-positive tumors [59]. We have summarized the above-described tests in Table 1.

**Table 1:** Biomarkers using conventional methods and multigene classification tools for breast cancer.

Indication	IHC/FISH/RT-PCR-based tests	Ref.	Microarray-based tests	Ref.
Endrocine therapy	ESR1 * (I) (P) and PGR * (I) (P)		ESR1	[29]
			H:I ratio (tamoxifen)	[43]
			RecurrenceOnline	[40]
Targeted therapy	HER2 * (I) (F) (P)		HER2	[29]
			RecurrenceOnline	[40]
Grade	FoxTop (P)	[60]	MapQuant DX	[41]
Chemotherapy response	PAM50 (P)	[44]	MapQuant DX	[41]
	Oncotype DX (P)	[61]		
Prognosis	Oncotype DX (P) CURIO (I) Celera Metastasis Score (P) BreastOncPx (P)	[10] [26] [56] [57]	70 gene * RecurrenceOnline IGS HDP Rotterdam signature	[42] [40] [54] [55] [62]

* FDA approved diagnostic biomarkers, (I): IHC, (F): FISH, (P): RT-PCR, ESR1: Estrogen Receptor, PGR: Progesterone Receptor, HER2: Human Epidermal Growth Factor Receptor 2.

## 5. Solitary Genes of the Signatures

The value of single genes within gene expression signatures remains an open question. We will discuss some studies in which biomarker candidate discovery performed by transcriptome analyses was followed by *in vitro* investigation and clinical validation of its prognostic or predictive power in independent patient cohorts. 

For endocrine therapy response, MYC has been identified as a key molecule in estrogen independent growth utilizing gene expression signature of long term estrogen-deprived cells. Its potential to predict poor outcome for patients following tamoxifen administration was then evaluated in three independent patient cohorts including 164, 181, and 298 patients [63]. Another biomarker candidate for endocrine therapy response prediction is PUMA. *In vitro* results indicated the role of PUMA in apoptotic dysregulation which could have an impact on progression and therapy response. Its association with breast cancer specific death and worse outcome of tamoxifen treated patients was confirmed in the case of 148 and 201 patients, respectively [64].

Similar studies were also conducted for chemotherapy response. The PSMB7 gene was discovered as a new biomarker candidate for anthracycline resistance by comparing drug resistant and -sensitive cell lines. Its driver role in doxorubicin resistance was assessed by a combination of gene silencing and drug treatment. Its high expression was linked to unfavorable prognosis in 1,592 breast cancer patients [65]. Recently, new biomarker candidates including the HMGA2 gene of the beta-catenin signal transduction pathway were discovered by ChIP analysis of 55 patients. Its expression predicted relapse-free survival and metastasis in 82 triple negative breast cancer patients [66]. HMGA2 was previously linked to multidrug resistance by a microarray analysis of cancer cell lines [67].

The expression of the IGFBP3 receptor had a strong prognostic value for predicting relapse-free survival time in 478 basal type breast cancer patients. *In vitro* results also show that IGFBP3 has a significant role in cell proliferation control [68]. The CDK8 gene was identified as a mediator of chemotherapy-induced tumor-promoting paracrine activities in CMV-GFP and NFKB-GFP reporter cell lines. The association between CDK8 and survival was further confirmed in 2897 breast cancer patients [69]. The ATIP3 was identified as predicting overall survival in metastatic breast cancer patients using microarray data of 150 patients and was validated in 162 samples. The role of ATIP3 was investigated *in vitro* via silencing and *in vivo* via mouse experiments showing that ATIP3 delays metastatic progression and limits the growth of metastases [70]. Many of these genes could be potential targets of personalized treatments of metastatic breast cancer. However, to date these predictor candidates have not yet made their way into therapeutic protocols, mostly because they are limited to selected drugs while current systemic protocols use a multi-target approach employing several systemic therapy agents sequentially or simultaneously. Most probably findings related to single genes will be even more valuable for the pharmacological drug research when utilizing new molecular targets.

## 6. Validation Studies

The publication of numerous microarray datasets in large-scale public repositories including GEO [71] and EGA [72] enables the cross-validation of findings obtained in different studies. By integrating several independent datasets into a single database it is possible to investigate sub-cohorts which could not include sufficient number of patients by evaluating any of the original datasets alone. Such an integrated online database for evaluating survival-associated biomarkers has already been constructed for breast cancer [73]. In the most recent version of the tool, in addition to single genes combination, signatures can also be evaluated in one analysis [74]. 

**Figure 1:**
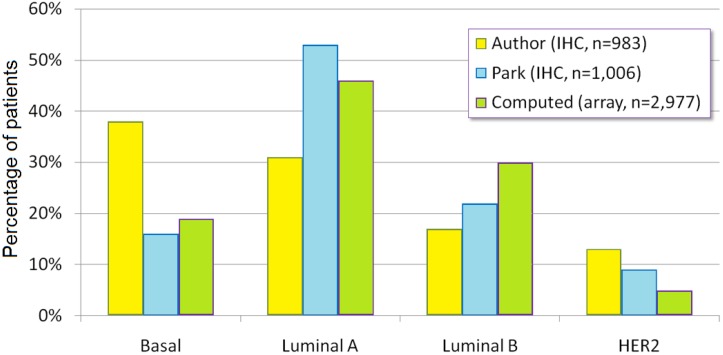
Reproducibility of the molecular subtypes in three different approaches. Author: as published in GEO by the authors of six datasets. Computed: distribution for all arrays in the database of the Kaplan-Meier plotter [82]. The receptor status determination was performed using Affymetrix HGU133x platforms by using a cutoff of 1,150 (MAS5 normalized value) for HER2 and 500 for ESR1. Park: as in reference [75].

An important issue in such meta-analyses is the reproducibility of previously published, conventional, pathology based biomarkers and subtypes. An example for these is the above discussed molecular subtype. In a recent large scale analysis Park *et al.*, established the subtype distribution in 1,006 patients [75]. In GEO, six datasets are available with determined molecular subtypes, including GSE1456 [76], GSE21653 [77], GSE25066 [78], GSE20711 [79], GSE31519 [80] and GSE17907 [81]. The re-computation of subtypes in these datasets is possible with the use of current StGallen guidelines and validated probe sets of Affymetrix HGU133x arrays (Table 2). By comparing the author reported prevalence of subtypes in the six independent datasets, the immunhistochemistry-results of Park *et al.* [75], and the re-computed subtypes for patient samples included in the Kaplan-Meier plotter [73], the highest concordance can be observed between the array-based and the IHC based determinations (see Figure 1). In these, basal tumors show the highest overlap and luminal B tumors show the highest discordance. At the same time, in the author-reported prevalence basal tumors are massively over-represented. The most probable reason for this is the predominant investigation of triple negative breast cancers as these patients have the worst prognosis among all molecular subtypes.

**Table 2:** The determination of molecular subtypes can be performed using Affymetrix microarrays and the appropriate classification by using the expression of three genes. The clinical difference between the two Luminal B cohorts is still not settled. Probe: Affymetrix HGU133A or HGU133plus2 arrays, N.R.: not relevant ESR1: Estrogen Receptor, HER2: Human Epidermal Growth Factor Receptor 2, MKI67: antigen identified by monoclonal antibody Ki-67.

Gene	Probe	Cutoff	Basal	Luminal A	Luminal B		HER2 positive
**ESR1**	205225_at	500	Low	High	High	High	Low
**HER2**	216836_s_at	4800	Low	Low	Low	High	High
**MKI67**	212021_s_at	470	N.R.	Low	High	N.R.	N.R.

A related question is dichotomization—in other words identification of the optimal cutoff points to determine two cohorts using continuous gene expression data. Researchers often employ packages like SPSS (SPSS Inc., Chicago, IL, USA), GraphPad Prism (GraphPad Software Inc., La Jolla, CA, USA) or WinStat (R. Fitch Software, Bad Krozingen, Germany) to correlate biomarkers with the outcomes or survival. Unfortunately, neither of these packages includes algorithms for cutoff optimization. For the generation of Figure 1, we used established cutoffs for ESR1 and HER2 which have been extensively validated [29,40]. For the proliferation marker MKI67, the median expression of the entire database was used. A more advanced determination of the optimal cutoff is possible by employing online-accessible algorithms specifically designed for cutoff determination [83]. 

The generated results of classification using multigene signatures are on different scales for 3-category classifiers (for example Oncotpye DX, RecurrenceOnline) and 2-category classifiers (for example Mammaprint, Genomic Grade Index). It is difficult to make comparisons between 3- and 2-category classifiers without biasing against the latter as the application of an intermediate outcome can significantly improve the correlation when only the high- and low-risk cohorts are compared. Although these statistical issues influence the performance of individual tests, they do not cast doubt on the overall performance of the developed diagnostic tools.

## 7. Conclusions

In our review, we summarized the current state of microarray-based advances for breast cancer pathological diagnosis. The true strength of multigene assays lies in the easily accessible independent validation. At present, there are tools available which integrate various diagnostic analyses into one test [40]. Multigene tests have already made their way into the diagnostic procedure and this process can be expected to speed up in the near future.
